# Trade-Off between Adsorption and Regeneration in Functional Metal–Organic Frameworks for Atmospheric Water Harvesting: A Multiscale Modeling Approach

**DOI:** 10.1021/acsami.5c25373

**Published:** 2026-03-04

**Authors:** Mehrzad Arjmandi, Mohamed Khayet, Takeshi Matsuura

**Affiliations:** † Department of Structure of Matter, Thermal Physics and Electronics, Faculty of Physics, 16734 University Complutense of Madrid, Avda. Complutense s/n, 28040 Madrid, Spain; ‡ Madrid Institute for Advanced Studies of Water (IMDEA Water Institute), Avda. Punto Com N◦ 2, 28805 Alcalá de Henares, Madrid, Spain; § Department of Chemical and Biological Engineering, University of Ottawa, 161 Louis Pasteur Private, Ottawa, Ontario K1N 6N5, Canada

**Keywords:** metal docking, metal–organic framework, atmospheric water harvesting, density functional theory, Grand Canonical Monte Carlo, Kinetic Monte Carlo, molecular dynamics

## Abstract

Understanding the trade-off between water adsorption and regeneration efficiency is essential for the rational design of functional metal–organic frameworks (MOFs) for atmospheric water harvesting (AWH). In this work, Cu-halide-functionalized MOF-303 is employed as a representative case study. A multiscale modeling framework combining Grand Canonical Monte Carlo (GCMC), Kinetic Monte Carlo (KMC), Density Functional Theory (DFT), and Molecular Dynamics (MD) simulations, along with a time-dependent thermodynamic analysis, is used to evaluate the impact of different Cu-halide ligands (F, Cl, Br, and I) on water adsorption–desorption behavior. Model accuracy is validated through comparison with previously reported experimental data from the literature for pristine MOF-303, showing good agreement between simulations and published experimental results. The results indicate that Cu–F@MOF-303 exhibits strong electrostatic interactions, leading to high water uptake and rapid adsorption kinetics, but at the expense of higher regeneration temperatures. In contrast, Cu–Cl@MOF-303 achieves a more balanced performance, offering moderate adsorption capacity with comparatively energy-efficient regeneration. Cu–Br@MOF-303 and Cu–I@MOF-303 enhance water uptake at low relative humidity (<20%) but require higher energy input for water release. Overall, this study demonstrates how targeted functionalization governs the adsorption–regeneration trade-off in MOFs and provides guidance for the sustainable design of AWH materials under diverse environmental conditions.

## Graphical abstract

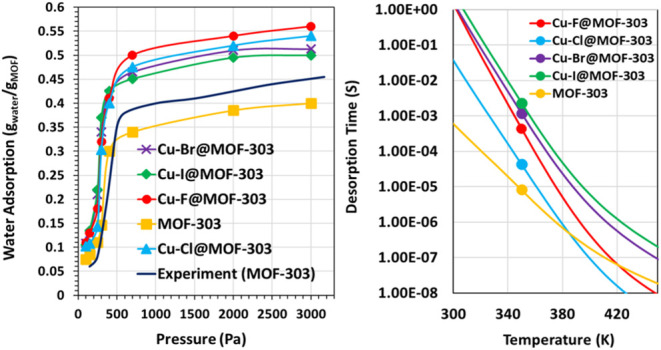

## 1 Introduction

With the intensifying impacts of climate change, rapid urbanization, and escalating global water demand, the availability of clean and drinkable water faces unprecedented stress.^1^ This growing crisis, particularly pronounced in arid and semiarid regions, has prompted the urgent need for innovative water generation strategies that do not rely on conventional hydrological resources.^2^ Among the emerging technologies, sorption-based atmospheric water harvesting (SAWH) has attracted substantial attention due to its passive operation, low energy consumption, and potential for decentralized water production.^3–5^ SAWH systems exploit the diurnal humidity cycle by adsorbing atmospheric water vapor during nighttime when relative humidity (RH) is high and releasing it during the daytime upon mild heating (Figure S1). A critical factor dictating the success of SAWH is the performance of the sorbent material in terms of capacity, uptake kinetics, regeneration efficiency, and long-term structural stability.^6^

Metal–Organic Frameworks (MOFs) have gained prominence as exceptional adsorbents for SAWH due to their remarkable surface area, tunable porosity, and versatile chemical functionality.^6^ Composed of metal ions or clusters coordinated by organic ligands, MOFs exhibit highly porous structures that provide extensive adsorption sites for water molecules.^7–9^ Among the various MOFs studied for water harvesting, MOF-303, formulated as [Al­(OH)­(PZDC)] with PZDC^2–^ representing 1-*H*-pyrazole-3,5-dicarboxylate, stands out due to its rod-like secondary building units (SBUs) formed by alternating cis–trans corner-sharing AlO_6_ octahedra connected through H_2_PZDC linkers.^10^ This framework features one-dimensional hydrophilic channels approximately 6 Å in diameter, which facilitate rapid water diffusion and high uptake capacity.^10^ MOF-303 demonstrates outstanding hydrolytic stability, maintaining its structure after more than 150 adsorption–desorption cycles, and shows water uptake capacities up to 0.48 g g^–1^ under RH = 20–40% at 303 K.^11–13^ Its practical applicability has been demonstrated by its integration into water harvesting devices that produce up to 1.3 L kg^–1^ day^–1^ under indoor conditions (32% RH, 300 K) and 0.7 L kg^–1^ day^–1^ in desert-like environments such as the Mojave Desert (10% RH, 300 K).^13^

Molecular-level engineering of MOFs has opened avenues for tuning their adsorption properties to better suit specific environmental conditions.^14,15^ Functionalization of MOFs, particularly through the incorporation of external metal ions, has been explored to improve adsorption capacity, selectivity, and stability.^16,17^ Metal docking strategies generally involve either coordination of metal ions to the SBUs or anchoring onto the organic linkers, often facilitated by linkers bearing chelating groups such as bipyridyls or through postsynthetic modifications.^17^ This structural engineering approach has been successfully applied to MOF-303, where precise postsynthetic metalation enabled the creation of functionally enhanced frameworks.^18^ By utilizing the spatial arrangement of uncoordinated nitrogen atoms along the Al–O rod-shaped SBUs, monovalent metal ions such as Cu­(I) and Ag­(I) were site-specifically incorporated with high efficiency.^17^ This metalated MOF-303 demonstrated outstanding performance in xenon gas separation, exhibiting high adsorption capacity and selectivity. Beyond gas adsorption, this strategy proved effective for aqueous applications; for instance, Ag^+^ incorporation improved nanofiltration performance in thin-film nanocomposite polyamide membranes.^19^ MOF-303 was also used as a scaffold to immobilize Cu species for capturing toxic radioiodine, achieving high uptake capacities and stability under humid and high-temperature conditions.^20^

Beyond metal docking, halide ions have recently gained attention as functional ligands to further modify metalated MOFs and improve their adsorption characteristics. Halides can alter the local electronic environment and pore chemistry, thereby influencing the interaction with adsorbates. Functionalization of MOFs with halide ions has been employed to enhance water adsorption by increasing hydrophilicity and creating additional binding sites. For example, incorporation of free halide ions into MOF-808 has been shown to prevent pore collapse and significantly enhance water uptake under low humidity by stabilizing the framework and introducing accessible sorption sites.^21^ Similarly, in the Ni_2_X_2_BTDD series (X: F, Cl, Br), the presence of fluoride ions enables strong hydrogen bonding with water molecules, resulting in notably higher water uptake capacity.^22^ In contrast, the larger bromide ions reduce pore volume, which facilitates earlier formation of hydrogen-bond networks.

Previous experimental studies on metal-docked MOF-303 have primarily focused on gas adsorption, separation, and pollutant capture applications, where the incorporation of monovalent metal ions such as Cu­(I) and Ag­(I) enhanced specific host–guest interactions. However, the impact of such metal docking on water adsorption behavior, hydrogen-bond network formation, and diffusion dynamics within the hydrophilic channels of MOF-303 have not been systematically evaluated. Given that water adsorption in SAWH systems is strongly governed by framework polarity, open metal sites, and cooperative hydrogen bonding, it is essential to understand how postsynthetic metal incorporation may alter these characteristics.

Similarly, although halide functionalization has demonstrated significant improvements in water uptake and framework stabilization in other MOF systems, analogous modifications have not been thoroughly explored for MOF-303. In particular, the potential of coordinated halide ligands to modulate the local electronic structure of metal centers while simultaneously introducing additional hydrogen-bonding sites within MOF-303 channels remains largely unexplored. Therefore, a systematic investigation of the combined metal docking and halide functionalization strategy is necessary to determine whether synergistic effects can be achieved for enhanced AWH performance.

In this study, we investigate the incorporation of Cu as a metal center alongside halide ligands (F, Cl, Br, I) coordinated to the framework, aiming to elucidate how these modifications affect the structural properties and water adsorption behavior of MOF-303. This work uniquely bridges this gap by systematically comparing the influence of different halide ligands (F, Cl, Br, I) coordinated to Cu centers on the water adsorption and transport behavior of MOF-303. We employ a comprehensive suite of molecular simulation techniques, including Grand Canonical Monte Carlo (GCMC) for adsorption isotherm prediction, Kinetic Monte Carlo (KMC) for adsorption kinetics, Density Functional Theory (DFT) for electronic structure analysis, and Molecular Dynamics (MD) for examining water transport properties within the pores. The computational framework employed in this study was validated against previously published experimental water adsorption isotherms of pristine MOF-303, ensuring the reliability of the simulation approach before extending the analysis to Cu-halide-functionalized systems. This multiscale approach provides fundamental insights to guide the rational design of high-performance MOFs tailored for real-world SAWH applications. Furthermore, the postsynthetic metalation and halide functionalization strategies discussed here are based on established and scalable synthetic procedures, suggesting potential for translating these computational insights into practical, larger-scale atmospheric water harvesting systems.

## 2 Models and Methods

### 2.1 Models

This study combined classical and quantum computational methods to examine the structural and adsorption characteristics of Cu-X@MOF-303 (X = F, Cl, Br, I) and pristine MOF-303. The crystal structure of MOF-303 was sourced from the Cambridge Crystallographic Data Centre (CCDC).^23^ A central portion of a supercell composed of four unit cells was selected as the simulation domain for MD, GCMC, and KMC simulations, as illustrated in Figure 1. For the electronic structure investigations, a simplified molecular structure was constructed to represent the local environment and was used in DFT calculations, as presented in Figure 2a–e. All computational procedures were performed on both the pristine MOF-303 and its Cu-metalated structures containing halogen atoms, including fluorine, chlorine, bromine, and iodine.

**Figure 1:**
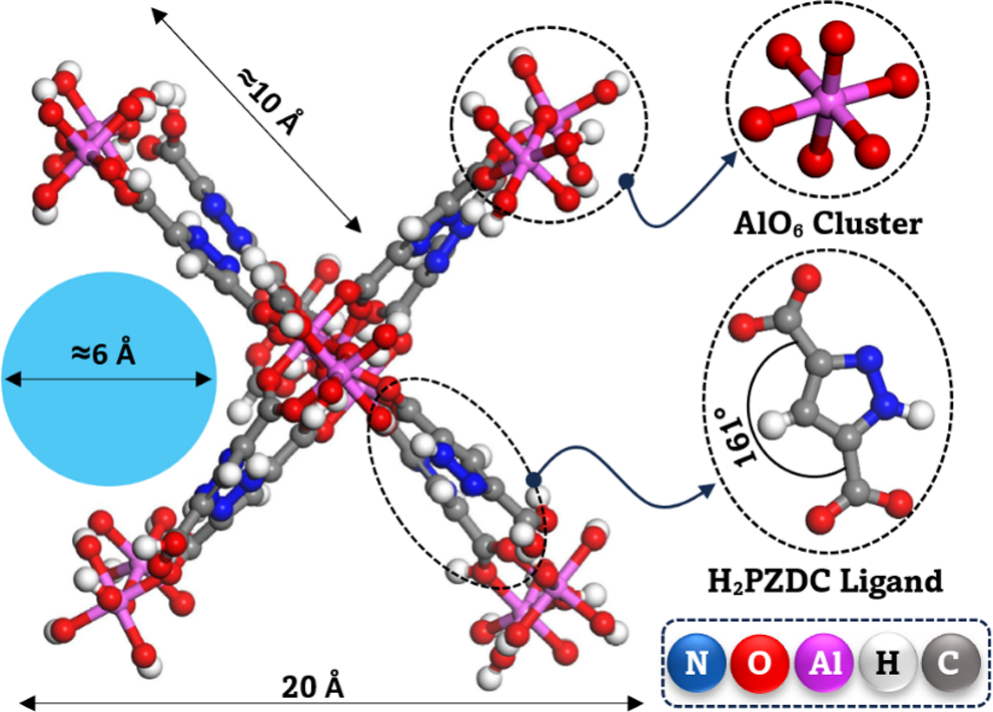
Structural model of MOF-303 used in simulations, illustrating the pore size. The ligand and metal cluster are also shown separately for clarity.

**Figure 2:**
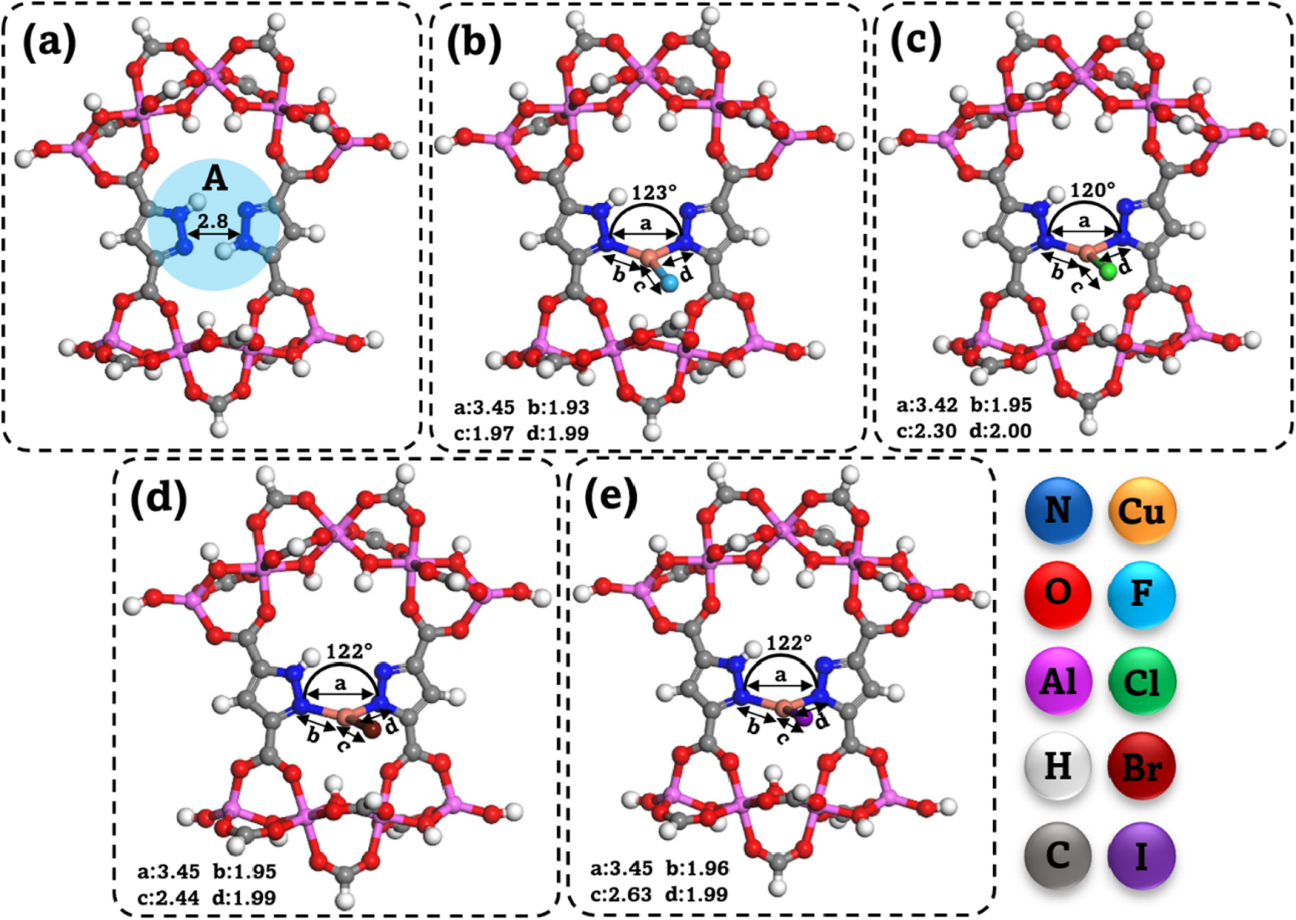
Simplified DFT models for (a) pristine MOF-303, (b) Cu–F@MOF-303, (c) Cu–Cl@MOF-303, (d) Cu–Br@MOF-303, and (e) Cu–I@MOF-303. Bond angles and distances (in Å) obtained from the optimized structures.

### 2.2 Ab Initio Calculations

To investigate the interaction behavior between water molecules and both the pristine and functionalized MOF-303 systems, a series of first-principles calculations based on DFT were conducted using the Gaussian 16W software package. Calculations were performed at the UB3LYP level using the SDD and LANL2DZ basis sets, providing a balance between computational efficiency and accuracy for transition-metal-containing MOF clusters.^24–27^ Since the UB3LYP functional is a spin-unrestricted formulation, the calculations inherently account for possible spin polarization of the Cu centers, ensuring that the adsorption and electronic properties correspond to the true ground-state electronic configuration. Terminal atoms were fixed at their crystallographic coordinates to preserve the rigidity of the original framework, while internal atoms were fully relaxed during geometry optimization. After structural optimization, water adsorption energies were estimated by calculating the single point energy as follows

1 $${E_{\mathrm{ads}}=E}_{\mathrm{MOF}+\mathrm{water}}-\left(E_{\mathrm{MOF}}+E_{\mathrm{water}}\right)$$

where *E*_MOF+water_ is the total energy of the water-MOF complex, and *E*_water_ and *E*_MOF_ represents the total energies of the isolated water and MOF molecule, respectively.^28,29^

Frontier molecular orbital analysis was performed to examine how halide substitution and water adsorption influence the local chemical reactivity and electronic symmetry. The energies and spatial distributions of the highest occupied molecular orbital (HOMO) and the lowest unoccupied molecular orbital (LUMO) were calculated within the framework of conceptual DFT. These orbitals are key indicators of the molecule’s electronic behavior, as their energy gap and localization patterns provide valuable insight into charge-transfer potential, polarizability, and the directional nature of host–guest interactions. To further assess the intrinsic electronic properties of the frameworks, Natural bond orbital (NBO) and Mulliken population analysis were performed to examine the internal charge distribution and the delocalization effects induced by Cu-halide incorporation. Additionally, the dipole moment (μ), was computed for the optimized MOF structures to quantify the influence of functionalization on molecular polarity.

### 2.3 Grand Canonical and Kinetic Monte Carlo Simulations

Although RASPA^30^ is a standard tool for Monte Carlo simulations in porous materials, this study adopted the LAMMPS package to facilitate a streamlined simulation environment where GCMC, KMC, and MD simulations could be integrated efficiently. This choice enabled capturing both equilibrium adsorption and time-dependent kinetics influenced by Cu–halide doping. LAMMPS offers flexible scripting capabilities, enabling hybrid MC-MD simulations and the implementation of residence-time-based kinetic models. To further support the validity of using LAMMPS for GCMC simulations, we note that previous studies have successfully implemented GCMC and MD protocols in LAMMPS for adsorption in MOFs and other porous materials.^31–35^ Furthermore, the implemented GCMC algorithm follows the standard Metropolis acceptance criteria and has been previously validated for adsorption studies in porous materials. The LAMMPS-based results also were benchmarked against RASPA simulations, showing good agreement.^35^ These validations demonstrate that the use of LAMMPS does not compromise accuracy while enabling seamless integration with MD and KMC simulations within a unified computational framework. This multiscale framework enabled simultaneous exploration of thermodynamic and kinetic contributions to water adsorption in pristine and functionalized MOF-303 under realistic conditions. A custom Python script automated the full simulation workflow, generating input structures under various scenarios. During GCMC runs, water molecules were permitted to undergo translation, rotation, reinsertion, and swap moves, while the MOF framework was held rigid throughout the simulations. The rigid framework approximation adopted in the present GCMC simulations (and consistently applied in the subsequent MD analysis) is justified on both experimental and methodological grounds. MOF-303 has been reported as a structurally rigid microporous sorbent exhibiting negligible hysteresis in its water sorption isotherm, indicating the absence of large-scale breathing or phase transitions during adsorption/desorption cycles.^13^ Structural investigations combining single-crystal X-ray diffraction and periodic DFT calculations have shown that water adsorption induces only minor local distortions of the linker orientations (∼0.4 Å) without global framework rearrangement.^36^ Furthermore, within the present modeling strategy, only the central portion of a supercell was considered for simulations. Allowing extensive structural flexibility in such truncated or constrained models would not realistically capture collective breathing modes and could introduce artificial boundary distortions. Therefore, even if moderate flexibility were present, treating the framework as rigid is both physically justified and methodologically necessary to ensure numerical stability and meaningful comparison among the functionalized systems.

In the GCMC simulations, water molecules were inserted randomly within the accessible pore volume of the MOF frameworks, ensuring no overlap with host atoms. Insertion and deletion moves were accepted or rejected according to the Metropolis criterion based on the chemical potential corresponding to the target pressure and temperature. Translation, rotation, swap, and reinsertion moves were allowed to enable thorough sampling of configurational space, including clustering and site-specific adsorption. For reinsertion, water molecules were randomly repositioned within the pore space, with repeated attempts if initial positions overlapped with the framework. Each system was equilibrated for 1 × 10^6^ MC steps, followed by 5 × 10^6^ production steps, resulting in over 10,000 distinct water configurations used for analysis. The average number of water molecules adsorbed in the pores under a given vapor pressure or RH was determined directly from the GCMC simulations. This equilibrium loading was then used as input for the subsequent MD simulations.

Interatomic interactions were described using a combination of 12–6 Lennard-Jones (L-J) and Coulombic potentials, following the equation^10,37^

2 $$U_{unbound}=4\varepsilon_{\mathrm{ij}}\left[{\left(\frac{\sigma_{\mathrm{ij}}}{r_{\mathrm{ij}}}\right)}^{12}-{\left(\frac{\sigma_{\mathrm{ij}}}{r_{\mathrm{ij}}}\right)}^{6}\right]+\frac{q_{i}q_{j}}{4\pi \varepsilon_{0}r_{\mathrm{ij}}}$$

3 $$\varepsilon_{\mathrm{ij}}=\sqrt{\varepsilon_{i}\varepsilon_{j}}$$

$$\sigma_{\mathrm{ij}}=\frac{\sigma_{i}+\sigma_{j}}{2}$$ 4

where ε_*ij*_ is the L-J well depth, σ_*ij*_ is the L-J collision diameter, *r*_*ij*_ is the distance between atoms *i* and *j*, *q* is the charge of each atom, and ε_0_ is the permittivity of vacuum. The Lorentz–Berthelot mixing rules were applied to define interaction parameters between nonidentical atom pairs. LJ interactions were truncated at 12 Å and long-range electrostatics were evaluated using the Ewald summation method with a precision of 10^–6^. Force field parameters for MOF-303 and its functionalized structures were taken from a combination of the DREIDING^38^ and Universal Force Field (UFF) force fields.^39^ Partial atomic charges were derived using the charge equilibration method based on DFT calculations with the Gaussian software package. Water molecules were modeled using the SPC/E model.^40^

To capture the dynamic aspects of water uptake, KMC simulations were performed, enabling time-resolved modeling of adsorption, desorption, and diffusion processes in the framework.^41,42^ The residence-time algorithm employed was based on the Bortz–Kalos–Lebowitz (BKL) approach, allowing simulation of rare events over extended time scales. This enabled exploration of kinetic phenomena such as surface hopping, clustering, and site-to-site transport with atomic specificity. Transition probabilities for elementary steps were derived from thermodynamic parameters obtained from DFT and GCMC simulations. The corresponding rate constants were calculated using the Arrhenius equation^43^

5 $$K_{i}=A_{i}\cdot \exp\left(\frac{-E_{i}}{\mathrm{RT}}\right)$$

where *A*_*i*_ is the pre-exponential factor, *E*_*i*_ is the activation energy, *R* is the universal gas constant, and *T* is the absolute temperature. This modeling strategy allowed for statistically meaningful sampling of rare events and provided a realistic picture of transport mechanisms and adsorption dynamics at the molecular scale.

### 2.4 Molecular Dynamic Simulations

Molecular dynamics (MD) simulations were carried out to explore the time-dependent behavior of water molecules confined within pristine and functionalized MOF-303 frameworks under ambient conditions. All simulations were performed using the LAMMPS software package in the canonical (NVT) ensemble, with the MOF frameworks treated as rigid and the water molecules modeled with full internal flexibility. The rigid-body approximation was adopted to prevent artificial distortions that could arise due to the truncated nature of the simulation cell, which includes only the central region of the supercell described in Section 2.1. This approach ensures numerical stability and preserves the crystallographic symmetry of the framework within the finite simulation domain. Moreover, the primary purpose of the MD simulations was to investigate the dynamics and interactions of confined water molecules rather than framework deformation. Previous computational studies employing larger simulation boxes have reported that introducing framework flexibility in MOF-303 leads to only minor changes in water adsorption and diffusion behavior,^44^ confirming that the rigid-body assumption remains physically consistent under comparable conditions.

The simulation box had dimensions of 28 × 28 × 28 Å^3^ under periodic boundary conditions, and a time step of 2.0 fs was used throughout the MD simulations. All force field parameters and interaction potentials were consistent with those described in Section 2.3. Temperature control throughout the simulation was maintained using the Nosé-Hoover thermostat.^45^ Simulation workflows, including trajectory generation and postprocessing, were fully automated using Python scripts. The MDAnalysis library was employed for trajectory analysis. Key structural and dynamic descriptors were extracted from the resulting trajectories.

To characterize molecular-scale interactions, radial distribution functions (RDFs) were computed based on atom-pair correlations between specific interaction sites. RDFs were calculated between oxygen atoms in water (OW) and copper centers (Cu), as well as between hydrogen atoms in water (HW) and halide ligands (F, Cl, Br, I). This analysis was conducted for both pristine and functionalized MOF-303 systems under two representative water vapor pressures, 250 and 700 Pa, corresponding to conditions before and after the sharp adsorption step, respectively. The RDF was calculated using the standard expression^46,47^

6 $$\mathrm{RDF}\left(r\right)=\frac{n_{r+\mathrm{dr}}}{4\pi r^{2}\rho \mathrm{dr}}$$

where *r* is the distance from a reference atom, *n*_*r*+*dr*_ is the number of atoms in the spherical shell between *r* and *r* + *dr*, and ρ is the average atomic density.

To further investigate water transport behavior within the confined environment of MOF-303, the mean square displacement (MSD) of water molecules was calculated for both pristine and functionalized frameworks. This analysis enabled quantitative assessment of translational mobility and revealed how structural modifications influence dynamic behavior. The MSD provides a statistical measure of the average squared distance that particles travel over time and is defined as^48,49^

7 $$\mathrm{MSD}\left(t\right)=\frac{1}{N}\left\langle \sum_{i=1}^{N}{\left|r_{i}\left(t\right)-r_{i}\left(0\right)\right|}^{2}\right\rangle$$

where *N* is the total number of water atoms considered, *r*_*i*_(0) and *r*_*i*_(*t*) denote the position vectors of atom *i* at the initial and later times, respectively, and the brackets represent the ensemble average.

The self-diffusion coefficient *D* of water molecules was obtained from the time-dependent MSD using Einstein’s relation^48^

8 $$D=\lim_{t\to \infty }\;\frac{\mathrm{MSD}\left(t\right)}{6t}$$

This coefficient quantifies the rate of molecular diffusion under confinement and was calculated separately for both frameworks at varying RH levels.

For MD simulations, the number of water molecules was fixed at the equilibrium loading obtained from GCMC, ensuring that the dynamic properties (RDF, MSD) reflect the same thermodynamic adsorption conditions.

### 2.5 Temperature-Dependent Desorption Behavior

To investigate the impact of temperature on the desorption process, adsorption energies were corrected for thermal effects by incorporating translational, rotational, and vibrational contributions^50,51^ as thermal corrections. This correction yields a temperature-dependent adsorption energy, *E*_ads_(*T*), which reflects the enhanced molecular motion and reduced binding strength at elevated temperatures. The corresponding desorption time, τ­(*T*), was estimated using the Polanyi–Wigner expression derived from transition-state theory (TST)^51–55^

9 $$\tau \left(T\right)=\vartheta_{0}^{-1}\cdot \exp\left(\frac{-E_{\mathrm{ads}}\left(T\right)}{k_{B}T}\right)$$

where *E*_ads_(*T*) is the temperature-dependent water adsorption energies, *T* is the temperature, and *k*_B_ is the Boltzmann constant. Here, *ϑ*_*0*_ denotes the attempt frequency, which represents the vibrational frequency of a water molecule in its adsorption site. It was estimated from the transition-state theory approximation ν_0_ *ϑ*_*0*_ = *k*_B_ *T*/*h* (where *h* is Planck’s constant), giving values on the order of 10^12^–10^13^ s^–1^ within the investigated temperature range.^56–59^ Because its temperature variation is small compared to the exponential Arrhenius dependence, in this study *ϑ*_*0*_ was assumed to be a constant value of 6 × 10^12^ s^–1^. Increasing temperature enhances molecular motion, weakening adsorption and accelerating desorption kinetics.

## 3 Results and Discussion

### 3.1 Water Adsorption Study

Figure 3 presents GCMC-simulated water adsorption isotherms for pristine MOF-303 and Cu-X@MOF-303 (X: F, Cl, Br, I), along with experimental data for MOF-303 from ref 60. for validation. As shown in Figure 3a, the simulated isotherm for MOF-303 reproduces the qualitative behavior observed experimentally: a gradual uptake at low pressures followed by a sharp increase near saturation. The underestimation in absolute uptake is attributed to structural simplifications in the model. Halide incorporation enhances the overall water uptake, with Cu–F@MOF-303 showing the highest saturation capacity, about 40% higher than the unmodified framework. Cu–Cl@MOF-303, Cu–Br@MOF-303, and Cu–I@MOF-303 follow in decreasing order, with improvements of roughly 35%, 28%, and 25%, respectively. This trend reflects increased hydrophilicity and the introduction of additional binding sites due to Cu-halide functionalization. Figure 3b, which highlights the low-pressure region (*P* ≤ 450 Pa), reveals a distinct pattern. Despite their lower saturated water adsorption capacities, Cu–Br@MOF-303 and Cu–I@MOF-303 exhibit even higher water uptake than Cu–F@MOF-303 in this regime. This behavior is attributed to the larger ionic radius of Br and I, which are expected to partially reduce the accessible pore volume and modify the internal pore environment. Previous experimental studies on Cu–Cl-functionalized MOF-303 systems have reported a reduction in pore size upon Cu–Cl docking.^17^ Considering the increasing ionic radius along the halide series (F < Cl < Br < I), a progressive narrowing effect is structurally plausible. Although pore size distribution (PSD) was not explicitly calculated in this study, the observed adsorption trend is consistent with confinement-enhanced water clustering under partially reduced pore volume conditions, which enables water molecules to initiate an extended hydrogen-bond network at lower relative humidity.^22^ Cu–F@MOF-303 also demonstrates superior uptake at low pressure compared to Cu–Cl@MOF-303, despite fluoride’s smaller size.^22,61^ This is explained by the strong hydrogen bonding interactions between water molecules and the highly electronegative fluoride ions, which promote early adsorption.

**Figure 3:**
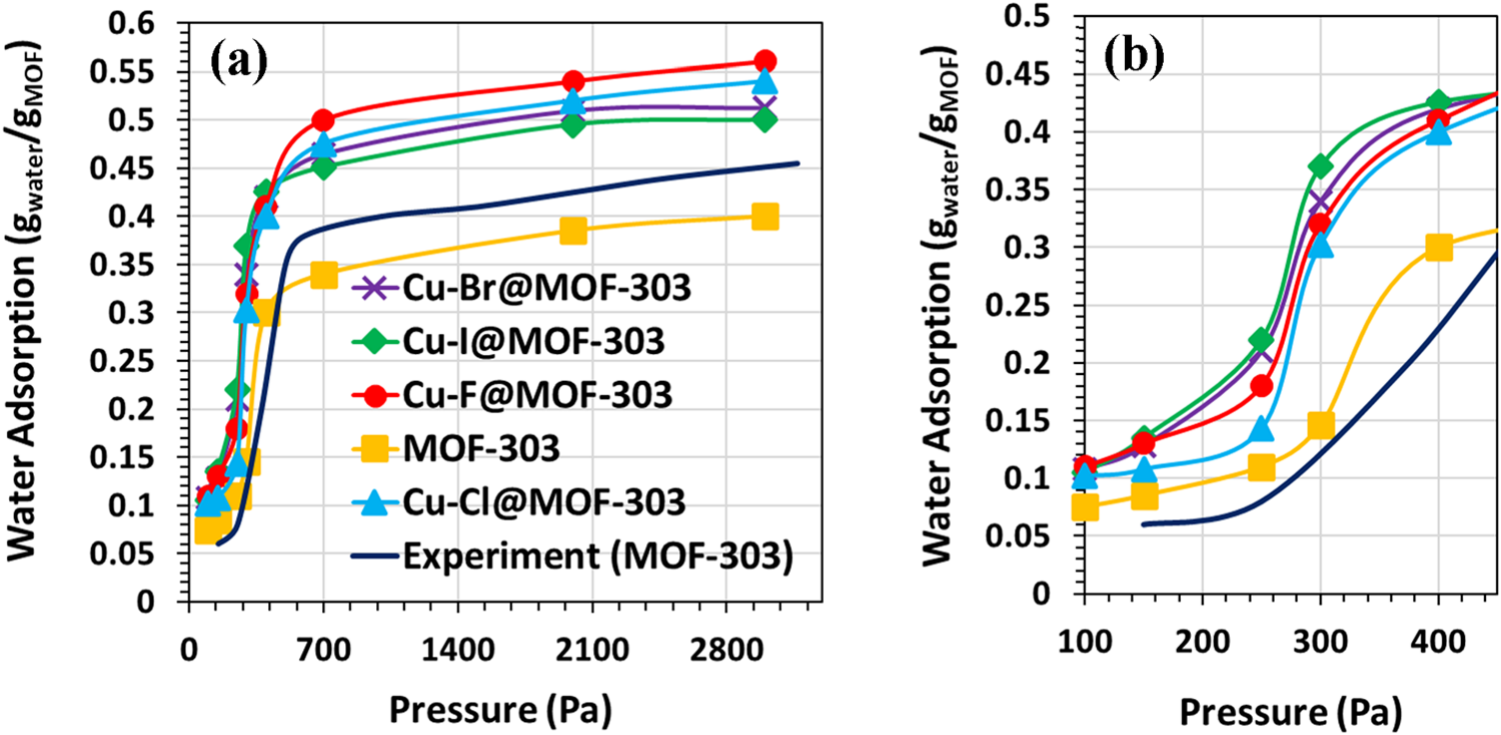
GCMC water adsorption isotherms for pristine MOF-303 and Cu-X@MOF-303 (X = F, Cl, Br, I): (a) full range; (b) low-pressure region (*P* ≤ 450 Pa), obtained from simulations at 298 K.

The results highlight the dual effect of halide type on water adsorption. Larger halides (Br, I) limit total capacity by occupying pore space but enhance uptake at low pressures by altering the adsorption mechanism. In contrast, smaller and more electronegative halides like F improve both low- and high-pressure performance through stronger specific interactions with water.

The water adsorption kinetics of pristine and Cu-halide functionalized MOF-303 structures were evaluated under two representative pressures (700 and 2000 Pa) using KMC simulations (Figure 4a–e). For the pristine MOF-303 (Figure 4a), saturation was reached in approximately 9 min at 700 Pa (∼22% RH) and 4 min at 2000 Pa (∼60% RH). These results are in qualitative agreement with previously reported experimental trends,^13^ which indicated full saturation at 20% RH after ∼10 min (and 5 and 3 min at 30% and 40% RH, respectively, at 303 K). We note that the experimental conditions differ slightly from our simulations (∼22% RH and ∼60% RH, at 298 K); therefore, the experimental data are cited for reference only, and the KMC results are not directly overlaid on the figure. This comparison supports the reliability of our kinetic model for the pristine system. All systems exhibit faster uptake rates at higher pressure, consistent with the expected pressure dependence of adsorption kinetics.

**Figure 4:**
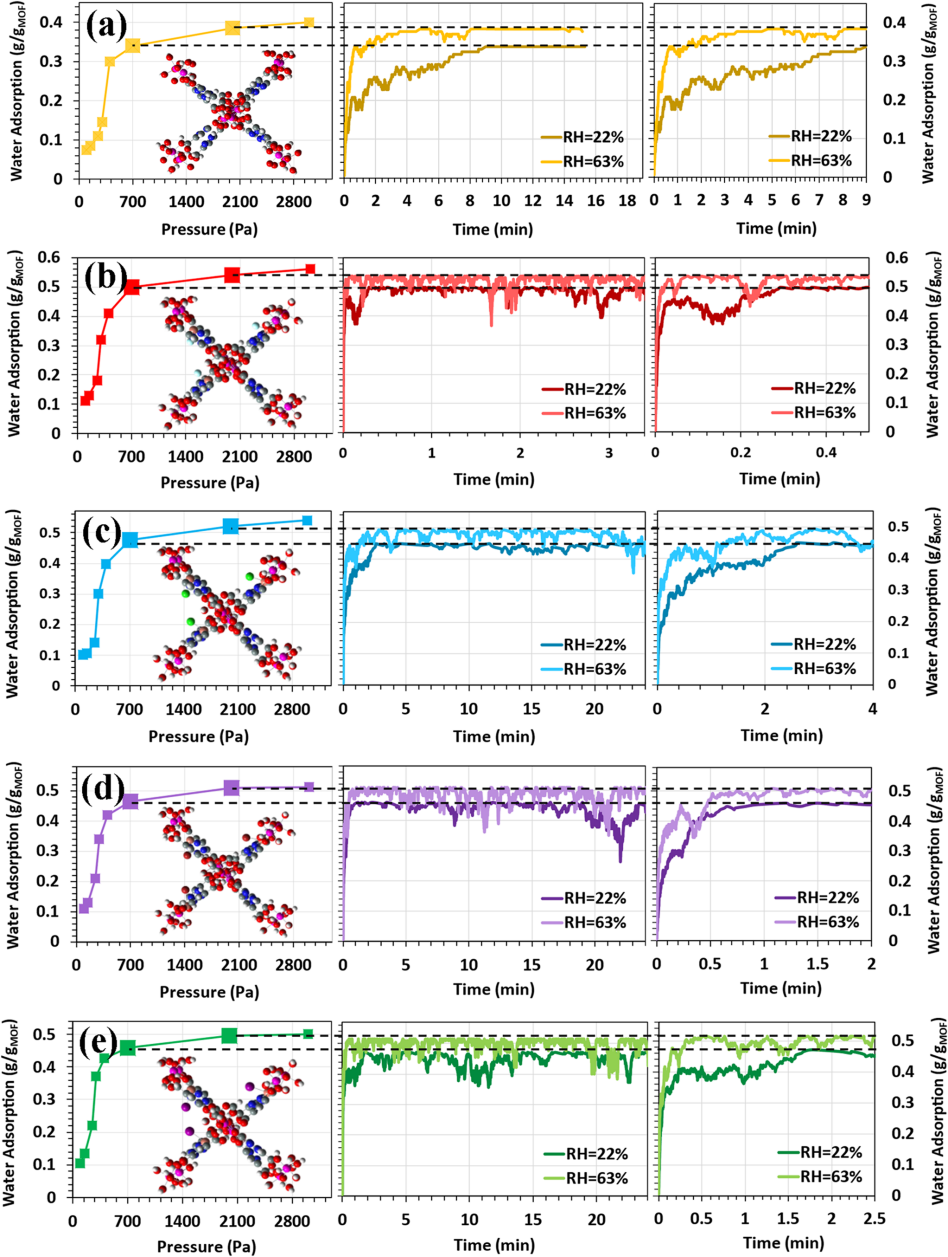
Water adsorption kinetics for (a) MOF-303, (b) Cu–F@MOF-303, (c) Cu–Cl@MOF-303, (d) Cu–Br@MOF-303, and (e) Cu–I@MOF-303 at 700 and 2000 Pa, obtained from simulations at 298 K.

Functionalization with Cu-halide species markedly enhanced water uptake kinetics across all conditions. Cu–F@MOF-303 displayed the most pronounced acceleration, achieving saturation within ∼18 s at 700 Pa and ∼5 s at 2000 Pa. This rapid response reflects the high hydrophilicity and strong hydrogen bonding capability of fluoride ions,^62^ which promotes faster nucleation and propagation of water clusters within the pores. Cu–Cl@MOF-303, while demonstrating the second-highest saturation uptake among the functionalized frameworks (as shown in Figure 3a), exhibited the slowest kinetic response, requiring ∼3 min at 700 Pa and ∼2 min at 2000 Pa. Furthermore, as observed in Figure 3b, its low-pressure isotherm slope was the lowest among the functionalized MOF-303 materials. Cu–Br@MOF-303 and Cu–I@MOF-303 showed substantial improvements, reaching saturation in under 1.7 min at 700 Pa and below 1 min at 2000 Pa.

Figure 5 illustrates the time-dependent behavior of the real-space correction function C­(r) for pristine (Figure 5a) and Cu-halide functionalized MOF-303 (Figure 5b–e) systems. This function quantitatively describes the local deviation of water density from the bulk average at different radial distances around adsorption sites, offering insight into the dynamic structuring of water during the adsorption process. At the early stages of adsorption, all systems display negative values of C­(r) near the framework surface, indicative of initial depletion zones where water molecules have not yet accumulated. As time progresses and adsorption advances, C­(r) increases and becomes positive, reflecting the gradual clustering of water molecules around active regions of the framework. Among the studied systems, Cu–F@MOF-303 exhibits the most rapid and pronounced increase in C­(r), confirming its strong local affinity for water and exceptional adsorption kinetics observed in KMC simulations (Figure 4b). This behavior is attributed to the high electronegativity and strong hydrogen bonding propensity of the fluoride ion, which promotes initial nucleation and accelerates water cluster formation.

**Figure 5:**
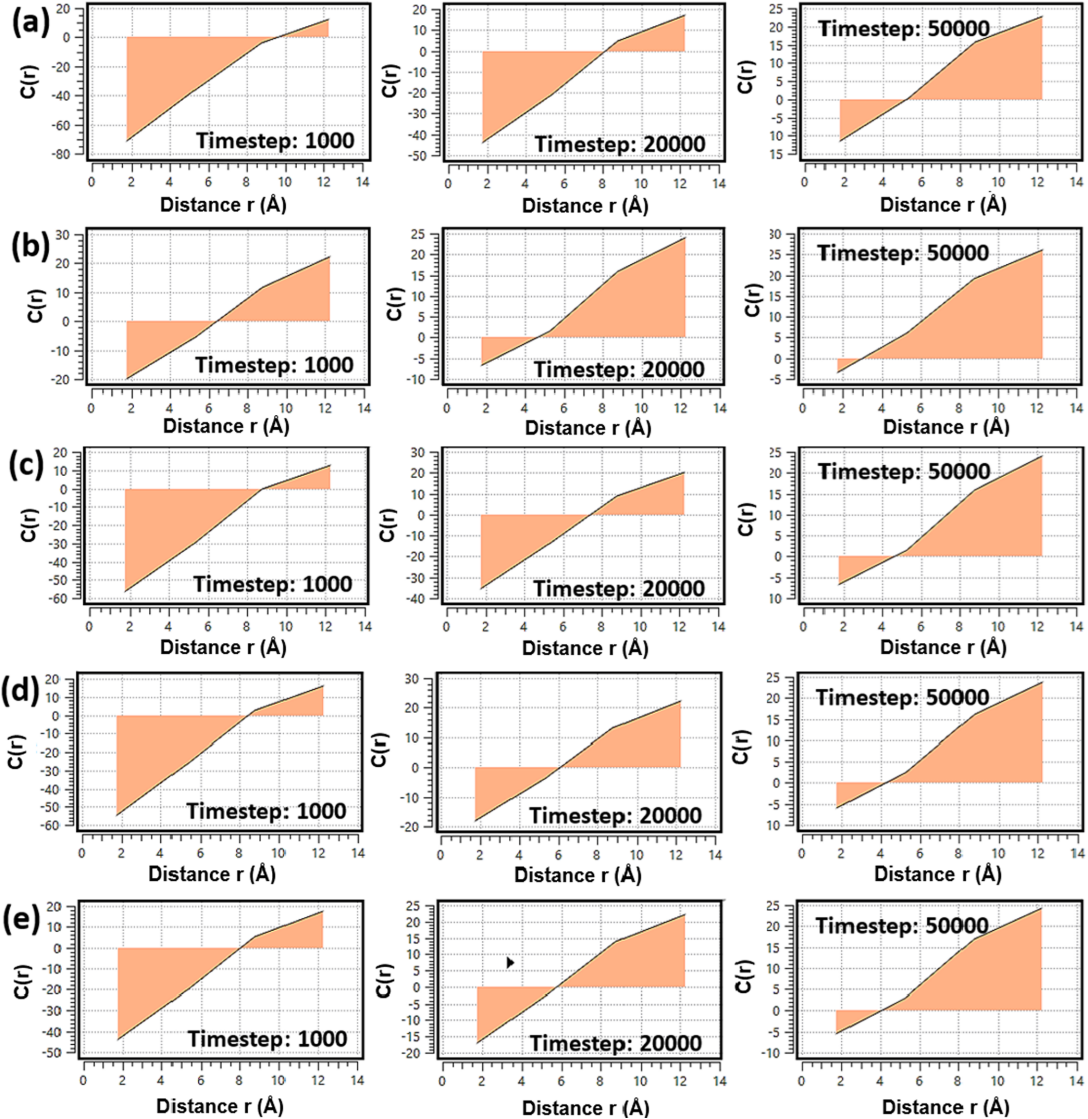
Time evolution of the real-space correction function, C­(r), for (a) pristine MOF-303, (b) Cu–F@MOF-303, (c) Cu–Cl@MOF-303, (d) Cu–Br@MOF-303, and (e) Cu–I@MOF-303.

In contrast, Cu–Cl@MOF-303 shows the slowest evolution in C­(r), with weaker and delayed positive shifts over time (Figure 5c), consistent with its comparatively sluggish uptake behavior reported in kinetic profiles. The moderate performance of Cu–Br@MOF-303 and Cu–I@MOF-303 is reflected in their intermediate C­(r) development curves (Figure 5d,e), where water density accumulation occurs faster than in the pristine or Cu–Cl systems, but more gradually than in Cu–F.

These findings reinforce the kinetic observations and support the notion that halide identity significantly influences the local hydration dynamics. Fluoride’s superior performance arises not only from favorable thermodynamic interactions (as shown in isotherms) but also from its ability to rapidly promote local structuring of water, an essential feature for efficient AWH operation under low-humidity conditions.

While the GCMC and KMC results generally align with experimental trends, some discrepancies in absolute water uptake values are observed. These differences can arise from structural simplifications in the models, neglect of framework defects, and limitations inherent to force fields and simulation time scales. To gain deeper insight into the observed variations in adsorption behavior across different pressure regimes, as well as to more precisely elucidate the underlying mechanisms governing the kinetic differences among functionalized frameworks, further investigations at the quantum level are required.

### 3.2 Structural and Electronic Characteristics

To enhance the understanding of the water adsorption mechanism, we supplemented isotherms obtained from GCMC and kinetic simulations based on KMC with quantum mechanical calculations using DFT. To gain initial insights, various configurations of water molecules placed on the studied frameworks shown in Figure 2 were examined, revealing that regardless of their initial positions, the water molecules tend to cluster ultimately in the same region “A” illustrated in Figure 2a. Table 1 presents the calculated water adsorption energies (*E*_ads_) for pristine MOF-303 and Cu-halid@MOF-303 systems. The pristine MOF-303 exhibits an adsorption energy of −84.77 kJ mol^–1^, which is in reasonable agreement with previously reported values obtained from DFT calculations.^10^ The differences observed arise mainly from variations in computational approaches. This comparison confirms the consistency and reliability of our DFT results. Notably, incorporation of Cu-halide ligands significantly enhances the water affinity of the frameworks. Among the halides investigated, Cu–F@MOF-303 shows the strongest adsorption energy of −120.02 kJ mol^–1^, followed by Cu–I@MOF-303 (−110.43 kJ mol^–1^), Cu–Br@MOF-303 (−109.12 kJ mol^–1^), and Cu–Cl@MOF-303 (−105.42 kJ mol^–1^). These results indicate that halide substitution modulates the host–guest interactions, presumably by altering the local electronic environment and polarization characteristics of the framework. Although the calculated adsorption energies for pristine and Cu-halide-functionalized MOF-303 (−84.77 to −120.02 kJ mol^–1^) are relatively high and exceed typical physisorption ranges, the adsorption remains fundamentally physical. The elevated energies arise from strong hydrogen bonding, enhanced electrostatic interactions, and confinement effects within the MOF pores. It should also be noted that DFT calculations consider static framework configurations and isolated water clusters, which tend to overestimate interaction energies relative to dynamic, thermally averaged systems. GCMC and KMC simulations confirm reversible adsorption behavior, consistent with physisorption. Therefore, despite high energies, no covalent bond formation occurs, and the adsorption mechanism is dominated by noncovalent, physical interactions. The high adsorption energy calculated for Cu–F@MOF-303 corresponds well with its highest water uptake capacity observed in GCMC simulations and its very fast adsorption kinetics. The superior adsorption kinetics of Cu–F@MOF-303 can be attributed to the strong hydrogen bonding between water molecules and fluoride ions, which promotes rapid nucleation and growth of water clusters within the pores. In addition, the highly electronegative F atoms enhance local polarization and electrostatic interactions, further accelerating water adsorption. In contrast, the low adsorption energy of Cu–Cl@MOF-303 is consistent with its low slope in the low-pressure region of the GCMC isotherm and its slowest adsorption rate among the functionalized structures. These observations suggest that the second highest saturation capacity of this structure is mainly due to the smaller size of the Cl ion (compared to Br and I), which leaves more pore space available.^22^ In addition, the faster water uptake and higher water adsorption at low pressure in Cu–Br@MOF-303 and Cu–I@MOF-303 compared to the Cu–Cl@MOF-303 (see Figure 3b) variant are in line with their stronger calculated adsorption energies. Although the Br and I ions reduce the available pore volume and lead to lower saturation capacities, the stronger interaction with water promotes earlier adsorption.^22^ To further elucidate the electronic factors influencing these adsorption behaviors, we analyze several quantum chemical parameters. The dipole moment (μ) is first examined as a key indicator of the framework’s polarity and its electrostatic response during water adsorption. Subsequently, molecular orbital analyses, including HOMO–LUMO mapping, along with charge distribution, are discussed to gain a comprehensive understanding of the electronic structure and interaction mechanisms.

**Table 1:** Computed Adsorption Energies of Water in Pristine and Cu-halid@MOF-303 Frameworks

adsorbent	*E*_ads_ (kJ mol^–1^)	ref
MOF-303	–71.86	^10^
MOF-303	–84.77	this study
Cu–F@MOF-303	–120.02	this study
Cu–Cl@MOF-303	–105.42	this study
Cu–Br@MOF-303	–109.12	this study
Cu–I@MOF-303	–110.43	this study

Table 2 summarizes the computed dipole moments (μ) of pristine MOF-303 and Cu-halide@MOF-303 systems before and after water adsorption, alongside the corresponding changes (Δμ). Prior to water adsorption, the intrinsic dipole moment exhibits a clear and progressive increase upon incorporation of Cu-halide groups. This trend strongly correlates with the intrinsic polarizability of the halide ligands, which increases in the order *F* < Cl < Br < I.^63^ Specifically, the pristine MOF-303 shows the lowest dipole moment (2.137 D), whereas Cu–I@MOF-303 reaches the highest value (8.784 D). This systematic enhancement of dipole moment reflects increased electronic asymmetry and local polarization induced by the heavier, more polarizable halide ions within the framework, thereby modulating its electrostatic landscape.^64^ Upon adsorption of water, the dipole moment responds variably depending on the halide identity. Functionalized systems with the more electronegative and less polarizable halide (Cu–F@MOF-303) demonstrate a notable increase in dipole moment (Δμ = +5.312 D), indicative of strong local polarization effects and enhanced directional host–guest electrostatic interactions. This can be attributed to the high electronegativity of fluorine^65^ which creates localized electric fields that promote substantial electronic reorganization upon water adsorption. Conversely, the systems functionalized with heavier and more polarizable halides, exhibit slight changes in dipole moment after water adsorption. The relatively small changes in dipole moment for the heavier halides (Cl, Br, I) indicate that the induced polarization upon water adsorption is more delocalized or involves compensating electronic rearrangements, possibly due to the more diffuse electron clouds of Cl, Br and I which alter the spatial distribution of charge upon adsorption. This subtle redistribution of charge affects the local electrostatic environment, which in turn modulates water adsorption behavior. Thus, Δ*μ* not only quantifies polarization changes but also provides insight into how the framework’s electronic response to water varies with halide identity, helping to rationalize the observed trends in adsorption energetics and kinetics. The overall trend in dipole moment magnitude and its variation upon water adsorption is in strong agreement with the calculated water adsorption energies, which follow the order Cu–F@MOF-303 > Cu–I@MOF-303 > Cu–Br@MOF-303 > Cu–Cl@MOF-303 > pristine MOF-303. This correlation confirms that local electronic polarizability and the resultant electrostatic environment are key determinants of water affinity in these materials.^63^ This indicates that the trend of μ after water adsorption directly reflects the water adsorption strength and kinetics: systems with larger μ after (e.g., Cu–F@MOF-303) exhibit stronger adsorption energies and faster uptake, whereas systems with smaller μ after (e.g., Cu–Cl@MOF-303) show weaker interactions and slower kinetics. Δ*μ*, representing the change in dipole moment upon adsorption, mainly captures the local polarization induced by water but does not strictly correlate with the overall adsorption strength, especially for heavier halides where polarization is more delocalized. This distinction clarifies the connection between electronic structure, adsorption energetics, and kinetic behavior. In summary, the increasing polarizability of the halide ligands (F < Cl < Br < I) enhances the intrinsic dipole moment of the framework before adsorption, while the dipole moment after adsorption follows the same order as the adsorption energy (Cu-F > Cu–I > Cu–Br > Cu–Cl > pristine). This consistent trend demonstrates that the electronic factors governing dipole formation ultimately control water affinity and adsorption kinetics in these systems. To further unravel the electronic factors governing these behaviors, subsequent analyses of charge density redistribution and frontier molecular orbitals (HOMO–LUMO) are essential, providing a nuanced understanding of how halide functionalization tunes the electronic and adsorption properties of MOF-303 frameworks.

**Table 2:** Computed Dipole Moments (μ) of Pristine and Cu-halid@MOF-303 Systems before and after Water Adsorption, along with the Corresponding Change (Δμ)

adsorbent	μ before adsorption (D)	μ after adsorption (D)	|Δμ| (D)
MOF-303	2.137	3.682	1.545
Cu–F@MOF-303	5.245	10.557	5.312
Cu–Cl@MOF-303	6.777	7.218	0.441
Cu–Br@MOF-303	7.937	7.841	0.096
Cu–I@MOF-303	8.784	8.417	0.367

Figure S2 illustrates the spatial distribution of the HOMO orbitals for pristine MOF-303 and Cu-halide@MOF-303 systems before water adsorption. After water adsorption, the corresponding HOMO orbitals are shown in Figure S3. Before adsorption, halide functionalization systematically affects orbital localization. In Cu–F@MOF-303, HOMO orbitals are delocalized across the framework, indicating a symmetric electronic environment and a moderate dipole moment. As the halide atoms become heavier from Cl to Br and I, the orbitals increasingly localize to one side, indicating greater electronic asymmetry and corresponding higher dipole moments as reported in Table 2. This reflects the increasing polarizability of heavier halides, which destabilize symmetry and intensify local electric fields.^63^ As shown in Figure S3, water adsorption leads to a significant reorganization of the orbital distributions, which become elongated in the horizontal direction. This is most evident in Cu–F@MOF-303, where strong hydrogen bonding and enhanced charge-transfer interactions occur. These findings align with the largest dipole moment increase (Δμ = +5.312 D) and the strongest adsorption energy, supporting the idea that local electrostatic effects primarily drive water binding. Cu–Cl@MOF-303, Cu–Br@MOF-303, and Cu–I@MOF-303 show more confined orbitals after adsorption with minimal interaction toward water, indicating weaker electronic coupling and less polarization of the guest molecule. This matches the smaller dipole changes and lower adsorption strengths observed. The more limited orbital engagement observed for the heavier halides suggests they create more diffuse binding sites, which somewhat reduces the thermodynamic favorability for water uptake. However, the heavier halides still enhance water adsorption, demonstrating their meaningful contribution to the overall interaction. The combined analysis of Figures S2 and S3 confirms that lighter halides, particularly fluorine, produce localized electronic “hot spots” that enable stronger and more directional water-framework interactions.

To gain a deeper understanding of the electronic influence exerted by halide substitution on the Cu adsorption site in MOF-303, NBO and Mulliken charge analyses were carried out and illustrated in Figures 6 and S4. The charge distributions reveal a clear bifurcation in behavior between the Cu–F system and the heavier halides, underscoring the unique role of fluorine in modulating local electronic properties. In the Cu–F@MOF-303 system, the Cu center exhibits the highest positive charge among all system studied (Mulliken: +0.307; NBO: +0.671), paired with fluorine’s high negative charge (Mulliken: −0.440; NBO: −0.659). This substantial charge separation generates a pronounced local electronic polarization at the adsorption site, which is directly correlated with the strongest electrostatic interactions and the highest water adsorption energies recorded. The significant difference in charge distribution around the Cu–F pair creates a highly polarized environment, fostering directional hydrogen bonding with adsorbed water molecules and consequently enhancing thermodynamic binding affinity. In contrast, the other halides (Cl, Br, I), induce noticeably lower positive charges on the Cu center, reflecting a diminished electron-withdrawing capacity compared to fluorine. Correspondingly, these halides bear moderately negative charges; however, their absolute values and resulting polarization effects are more similar to each other than to the Cu–F system. Given their comparable charge characteristics and reduced local polarization, these heavier halide systems collectively exhibit weaker electrostatic fields at the adsorption site, which is consistent with their observed lower water adsorption affinities. Fluorine’s exceptional electronegativity and resulting charge separation create a distinct electrostatic landscape that strongly influences guest–host interactions.

**Figure 6:**
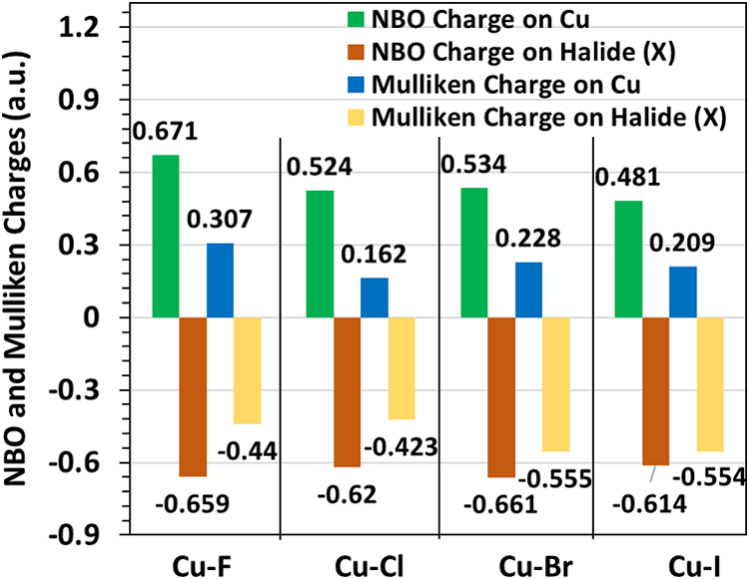
NBO and Mulliken charges of Cu and halide atoms in Cu-X@MOF-303 (X: F, Cl, Br, I).

### 3.3 Molecular-Level Study

To further elucidate the local interaction environment of water molecules within the functionalized MOF-303 frameworks, RDF analyses were performed for two key atomic pairs: copper and the oxygen atom of water (Cu–O­(W)) (Figure 7a,b) and halide and the hydrogen atom of water (Halide-H­(W)) (Figure 7c,b). These calculations were conducted at two RH levels, 8% and 22%, to investigate the spatial probability of water localization around the Cu, Halides, and finally within Zone A (see Figure 2). All cases exhibit a pronounced peak around 2.0–3.0 Å distance which corresponds to hydrogen bonds. Across all structures, a general trend is observed in which RDF intensities decrease as RH increases. This reduction reflects a diminished preference for water molecules to localize near specific adsorption sites at higher humidity. Instead, water begins to distribute more broadly throughout the porous framework, likely due to increased molecule–molecule interactions and reduced competition for individual binding sites. The RDF patterns are in strong agreement with the partial atomic charge distributions obtained from Mulliken and NBO population analyses, reinforcing the electrostatic origin of the observed structural trends. In Figure 7a,b, the RDFs for Cu–O­(W) interactions clearly distinguish Cu–F@MOF-303 from the other systems. At both 8% and 22% RH, this structure exhibits a sharper and more intense peak at shorter distances, indicative of stronger and more localized coordination between the Cu center and the oxygen atoms of water. This behavior is consistent with the higher positive charge by Cu in the fluoride-containing framework, which enhances its electrostatic attraction to water. In contrast, the RDF peak positions for the rest of the structures are shifted to slightly longer distances, indicating weaker Cu–O­(W) interactions and a more dispersed distribution of water molecules around the metal centers. This stronger interaction in Cu–F@MOF-303 provides a mechanistic explanation for its superior performance in previous simulations, including its highest adsorption energy, fastest uptake kinetics, and largest water capacity. The spatial proximity of water molecules to Cu facilitates early stage water clustering, which is essential for efficient adsorption under dry conditions.

**Figure 7:**
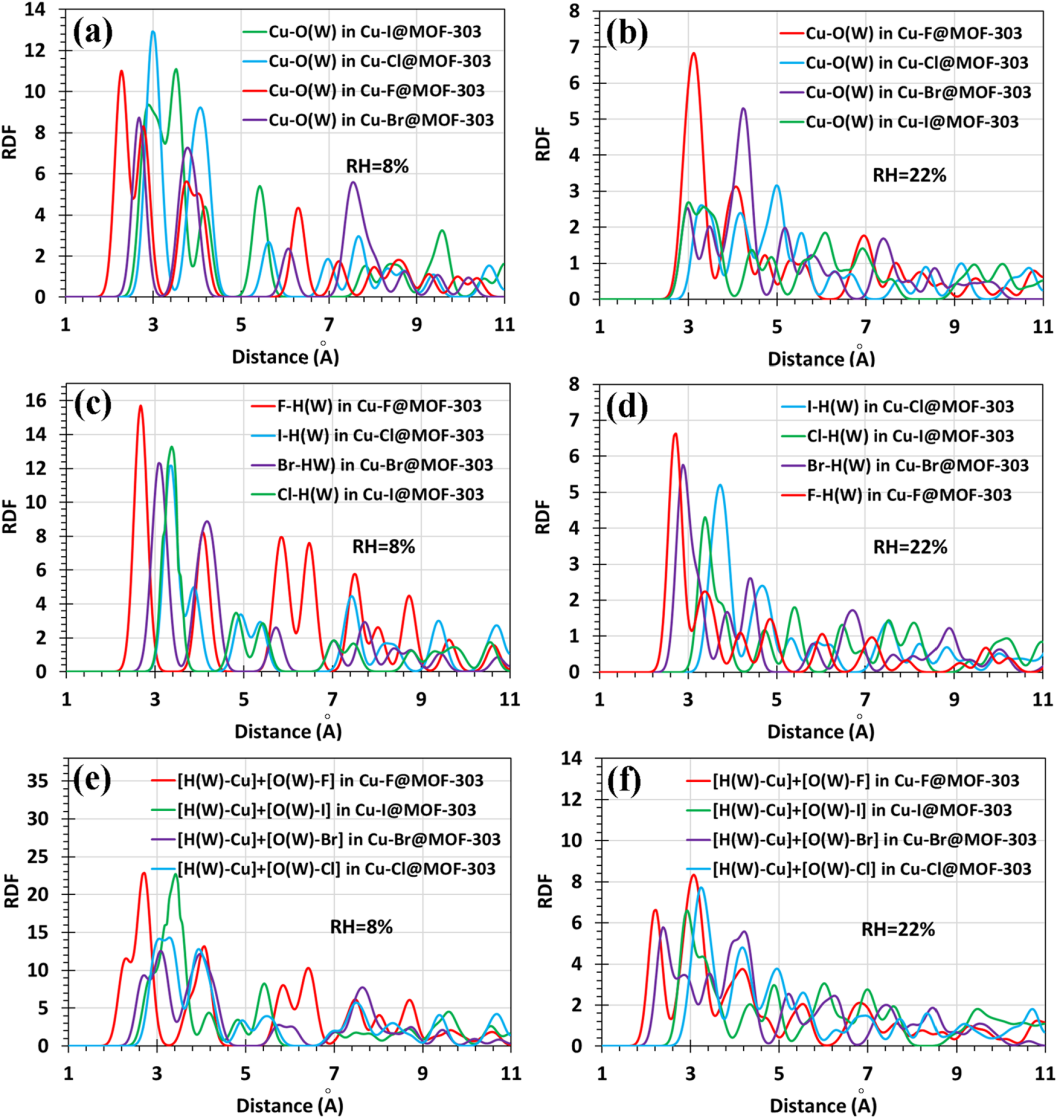
Cu–O­(w) RDFs at (a) 8% RH and (b) 22% RH for Cu-X@MOF-303 (X: F, Cl, Br, I) at 300 K. X-H­(w) RDFs at (c) 8% RH and (d) 22% RH for Cu-X@MOF-303 (X: F, Cl, Br, I) at 300 K. Combined RDF analysis showing total water localization near Cu and X sites in Cu-X@MOF-303 at (e) 8% RH and (f) 22% RH at 300 K.

Figure 7c,d shows the RDFs between halide atoms (X: F, Cl, Br, I) and the hydrogen atoms of water. The results demonstrate that F and Br exhibit significantly sharper, more intense, and closer RDF peaks compared to Cl and I, indicating stronger interactions and a more localized coordination of water molecules near the halide. In Cu–F@MOF-303, the short F–H­(W) distance and pronounced RDF peak confirm the strong hydrogen bonds between fluoride and water. A similar, though slightly weaker, feature is observed for Cu–Br@MOF-303. These findings are consistent with the more negative partial charges of F and Br in Figure 6, which increase their ability to attract and stabilize water via hydrogen bonding. In Cu–I@MOF-303 and Cu–Cl@MOF-303 the first RDF peak occur at larger distances. The weaker electrostatic field around Cl and I, along with steric effects especially for I, reduces their ability to support localized water binding. These observations further explain the relatively slow kinetics and lower initial uptake observed in Cu–Cl@MOF-303, despite its high saturation capacity, which may arise primarily from pore accessibility rather than strong site-specific interactions. Figure 7e,f combines Cu–O­(W) and X-H­(W) RDF data to estimate the overall probability of water localization within Region A. The results show that Cu–F@MOF 303 consistently exhibits the highest probability density of water presence near Cu, confirming that this structure offers the most favorable local environment for water uptake. This observation directly correlates with its stronger adsorption energy and superior GCMC and KMC performance. Cu–Cl@MOF-303 exhibits the farthest RDF peak position in Region A, indicating that water molecules interact at longer distances compared to other functionalized structures. Despite the stronger adsorption energy of Cu–I@MOF-303 compared to Cu–Br@MOF-303, the RDF analysis reveals a larger peak distance between water molecules and the Cu and I atoms, suggesting weaker spatial proximity. This discrepancy suggests that spatial factors, such as the larger ionic radius of iodide, impose steric hindrance that limits the accessibility of water molecules to the adsorption site. As a result, despite its thermodynamic favorability, Cu–I@MOF 303 exhibits the lowest saturation capacity in GCMC simulations. RDF analysis reveals that both electrostatic charge distribution and steric effects jointly govern the interaction strength and spatial localization of water molecules in Cu-X@MOF-303.

To investigate the dynamic behavior of water molecules within the MOF structures, MSD profiles were calculated for pristine and Cu-X@MOF-303 (X: F, Cl, Br, I) systems at 300 K and RH = 22%, as illustrated in Figure 8a. The pristine MOF-303 exhibits the highest MSD slope, indicating enhanced water mobility due to weaker host–guest interactions in the absence of metal-halide functionalization. Among the modified structures, Cu–F@MOF-303 shows the lowest MSD slope, followed by Cu–I@MOF-303, Cu–Br@MOF-303, and Cu–Cl@MOF-303, respectively. This order aligns closely with the previously calculated adsorption energies, where stronger interactions correlate with reduced water mobility. These results confirm that higher binding strength, particularly in Cu–F@MOF-303, effectively immobilizes water molecules within the framework, limiting their diffusion and indicating stronger confinement in the adsorption region.

**Figure 8:**
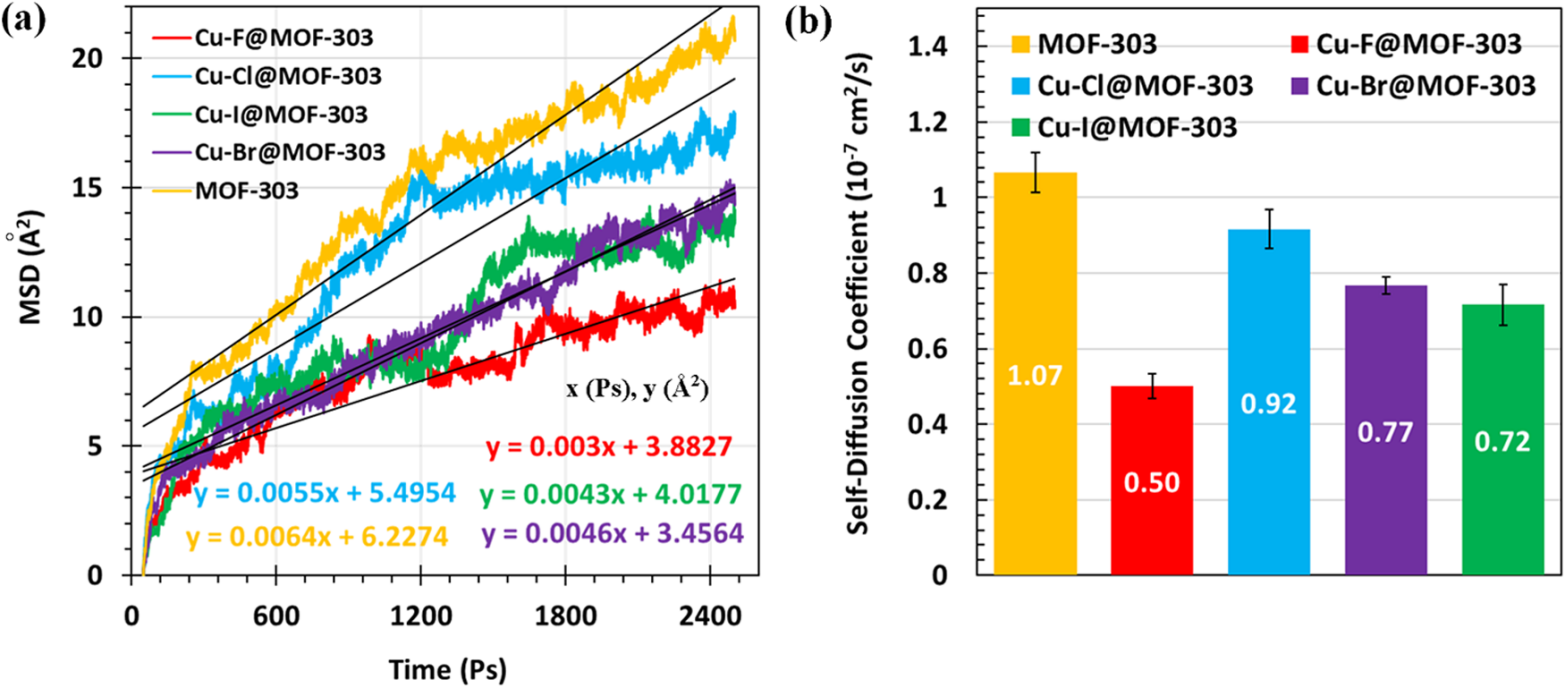
(a) MSD of water molecules in pristine MOF-303, Cu–F@MOF-303, Cu–Cl@MOF-303, Cu–Br@MOF-303, and Cu–I@MOF-303 (*T* = 300 K, RH = 22%). (b) Self-diffusion coefficients of water in pristine MOF-303 and Cu-X@MOF-303 (X: F, Cl, Br, I), as computed from MSD slopes at 300 K and RH = 22%.

Figure 8b shows the diffusion coefficients calculated from the linear regions of the MSD curves for pristine and Cu-X@MOF-303 (X: F, Cl, Br, I) systems. To verify the consistency of the calculated diffusion coefficients, the slopes of the MSD curves were re-evaluated over different linear fitting intervals (400–1100 ps, 1000–1700 ps, and 1700–3500 ps). The resulting diffusivity varied by less than ±8% for all MOF systems, confirming that the observed trends are not sensitive to the specific fitting range. The error bars in Figure 8b represent these variations, providing a quantitative estimate of the uncertainty associated with the diffusivity calculations. As expected, the pristine MOF-303 exhibits the highest diffusion coefficient, reflecting minimal interaction and confinement of water molecules in the unmodified framework. Among the functionalized structures, Cu–Cl@MOF-303 shows the highest diffusivity, while Cu–F@MOF-303 has the lowest. This trend is consistent with the adsorption energy results: stronger water-framework interactions in Cu–F@MOF-303 lead to more restricted molecular motion, whereas the weaker binding affinity in Cu–Cl@MOF-303 allows for enhanced water mobility. These results confirm the inverse relationship between adsorption strength and diffusivity, highlighting the trade-off between strong water binding for capacity and mobility constraints that may affect release dynamics in practical applications.

### 3.4 Desorption Study

Understanding the desorption behavior of water molecules is essential for evaluating the practical performance of MOF-based materials in AWH applications. Effective materials must combine high and fast water uptake with energy-efficient desorption at moderates. If desorption requires excessively high thermal input, the operational feasibility and sustainability of the system are compromised.

Figure 9a–e show the time-dependent fluctuation of adsorption energies for MOF-303, Cu–F@MOF-303, Cu–Cl@MOF-303, Cu–Br@MOF-303, and Cu–I@MOF-303, respectively, at various temperatures (100, 200, 350, 500, and 800 K). These time-resolved energy profiles are necessary to evaluate the dynamic stability of the adsorbed water molecules at finite temperatures. While static DFT adsorption energies provide interaction strength at 0 K, Figure 9a–e reveal how thermal motion affects the stability of the adsorbed state over time. Larger fluctuation amplitudes indicate increased dynamic instability and a higher probability of thermally activated desorption. Therefore, these figures provide microscopic insight into how temperature perturbs the adsorption equilibrium before calculating averaged thermodynamic quantities. In all three cases, the amplitude of fluctuations increases with temperature, indicating greater dynamic instability at higher thermal energy. Furthermore, the metalated structures exhibit overall higher fluctuation amplitudes than pristine MOF-303, with Cu–F@MOF-303 and Cu–Cl@MOF-303 showing the most pronounced variations across all temperatures.

**Figure 9:**
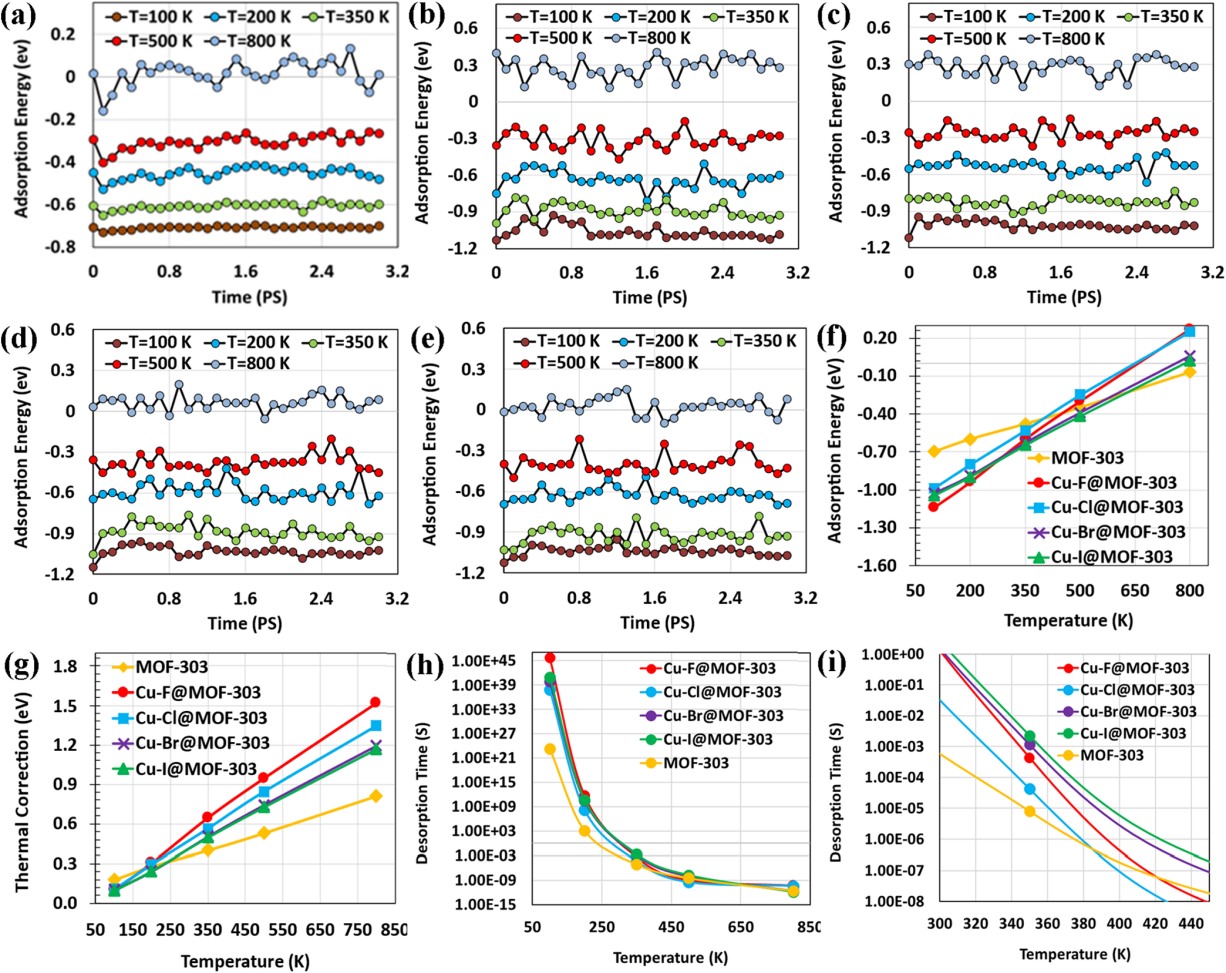
(a–e) Time-dependent fluctuations in adsorption energies at various temperatures (100–800 K) for MOF-303, Cu–F@MOF-303, Cu–Cl@MOF-303, Cu–Br@MOF-303, and Cu–I@MOF-303, respectively. (f) Average adsorption energies of H_2_O across the same temperature range for all three structures. (g) Corresponding thermal corrections of adsorption energies. (h) Estimated temperature-dependent desorption time constant (τ) for pristine MOF-303 and Cu-X@MOF-303 (X: F, Cl, Br, I) over the full range of 100–800 K and (i) focused view between 300 and 450 K.

Figure 9f shows average temperature-dependent adsorption energies of H_2_O on pristine MOF-303 and Cu-X@MOF-303 (X: F, Cl, Br, I) were computed over a temperature range of 100–800 K. As the temperature increases, the adsorption energies for all systems become less negative, indicating a progressive reduction in water affinity. Unlike Figure 9a–e, which illustrate instantaneous fluctuations, Figure 9f provides the averaged adsorption energies as a function of temperature, enabling direct comparison of thermal stability among different functionalized frameworks. This figure quantitatively demonstrates how halide identity controls the temperature sensitivity of water binding. Among all structures, pristine MOF-303 exhibited the least variation in adsorption energy with temperature, reflecting more stable interactions across the thermal range. In contrast, Cu–Cl@MOF-303 and especially Cu–F@MOF-303 displayed the steepest decrease in adsorption energy, suggesting that although these structures exhibit stronger binding at lower temperatures, they are more sensitive to thermal activation, which could facilitate desorption at elevated temperatures. Cu–Br@MOF-303 and Cu–I@MOF-303 showed intermediate behavior; more temperature-sensitive than pristine MOF-303 but less so than the fluorinated and chlorinated variants. This behavior implies a tunable balance between water retention and release, with halide identity directly influencing thermal desorption profiles.

Thermal correction values for adsorption energies were also computed and are illustrated in Figure 9g. Figure 9g is essential to separate purely electronic adsorption energy (0 K DFT value) from thermal contributions. By quantifying the thermal correction, we clarify how much of the adsorption strength is retained under realistic operating temperatures, which is critical for evaluating regeneration performance in AWH applications. For all five structures, thermal correction increases with temperature. However, the metalated structures exhibit steeper slopes and greater magnitudes of thermal correction compared to the pristine MOF-303. Among them, Cu–F@MOF-303 and Cu–Cl@MOF-303 show the highest degree of temperature-dependent change in thermal correction.

To evaluate the thermal desorption behavior of water in MOF structures, the desorption time constant τ was calculated based on the temperature-dependent adsorption energies using eq 5, and the results are shown in Figure 9h,i. Figure 9h,i translate the thermodynamic trends observed in Figure 9a–g into practical regeneration metrics, directly linking adsorption strength to desorption kinetics under realistic operating temperatures. As temperature increases, the τ values decrease across all systems, reflecting faster desorption kinetics at higher thermal input. This trend is clearly observed in Figure 9h. According to experimental data, desorption from MOF-303 typically occurs within the temperature range of 315 to 360 K,^13^ which is shown in Figure 9i. Within this range, Cu–Br@MOF-303 and Cu–I@MOF-303 exhibit significantly higher desorption time constants than the pristine and other functionalized frameworks, indicating stronger water retention under moderate heating. At 350 K, the τ values are 8.16 × 10^–6^ s for pristine MOF-303, 4.28 × 10^–5^ s for Cu–Cl@MOF-303, 4.36 × 10^–4^ s for Cu–F@MOF-303, 1.18 × 10^–3^ s for Cu–Br@MOF-303, and 2.29 × 10^–3^ s for Cu–I@MOF-303. Taking the behavior of pristine MOF-303 at 350 K as a reference, where water desorption occurs with a characteristic time constant of approximately 8.16 × 10^–6^ s, one can estimate the equivalent temperatures required for the functionalized materials to reach similar desorption performance. Based on this, Cu–Cl@MOF-303 would need to reach 363 K, Cu–F@MOF-303 around 376 K, Cu–Br@MOF-303 about 392 K, and Cu–I@MOF-303 approximately 398 K. Furthermore, at 363 and 376 K, the τ values for Cu–Cl@MOF-303 and Cu–F@MOF-303, respectively, match the desorption kinetics of pristine MOF-303 at 350 K. Based on the combined analysis of water uptake (GCMC, Figure 3), low-pressure adsorption, temperature-dependent adsorption energies, thermal corrections, and desorption time constants (Figure 9f–i), Cu–Cl@MOF-303 provides the best balance between adsorption performance and regeneration efficiency. While Cu–F@MOF-303 shows slightly higher water uptake, it requires higher thermal energy for desorption, whereas Cu–Br@MOF-303 and Cu–I@MOF-303 exhibit slower regeneration kinetics. These results emphasize that while functionalization enhances adsorption performance, it also increases the thermal energy required for regeneration, highlighting the need to balance water binding strength with energy-efficient release for real-world AWH applications.

## 4 Conclusions and Future Outlook

This study demonstrated that halide-functionalized Cu@MOF-303 frameworks can significantly enhance water adsorption performance for SAWH. Through a combination of GCMC, KMC, DFT, and MD simulations, we showed that halide identity strongly influences water uptake capacity, adsorption kinetics, dynamics, desorption behavior, and diffusivity. Among the studied materials, Cu–F@MOF-303 exhibited the highest uptake and fastest kinetics due to strong electrostatic interactions, though it required higher regeneration temperatures. Cu–F@MOF-303 is preferable for higher uptake near 20% RH if higher desorption temperatures are acceptable. Cu–Cl@MOF-303 presented a good trade-off between performance and energy demand, especially around 25% RH and above. Considering all factors influencing AWH performance, Cu–Br@MOF-303 and Cu–I@MOF-303 are suitable only when maximizing uptake below 20% RH is the primary goal and sufficient energy for desorption can be provided. Overall, the halide-functionalized Cu@MOF-303 structures offer a tunable balance between enhanced water adsorption and energy-efficient thermal desorption, demonstrating their practical applicability for real-world AWH systems. These findings emphasize the crucial influence of local polarization and charge redistribution in improving both water uptake capacity and adsorption kinetics, while also highlighting the significance of desorption behavior as a key factor in determining the practical applicability of MOFs for atmospheric water harvesting. These findings provide a general insight that can guide the rational design of other MOFs with tunable water adsorption properties. Although the present simulations were conducted under specific temperatures and RHs, the observed trends in water adsorption capacity, uptake kinetics, and desorption behavior are primarily dictated by the intrinsic structural and electronic features of the halide-functionalized Cu@MOF-303 frameworks. Therefore, these findings are expected to hold qualitatively across a broad range of typical AWH operating conditions, providing guidance for practical applications under varied environmental settings.

Potential limitations include the need for higher desorption energy for some materials and the lack of experimental validation at this stage. Future studies should explore experimental confirmation, long-term cycling stability, scalability, as well as integration of these MOF-based materials into practical AWH devices.

## Supplementary Material



## Notes

Schematic illustration of the SAWH process; spatial distribution of HOMO orbitals for pristine and Cu-X@MOF-303 systems before and after water adsorption; and NBO and Mulliken charge distribution analyses (Figures S1–S4) (PDF)
