# Supplementary material for: Trade-Off between Adsorption and Regeneration in Functional Metal–Organic Frameworks for Atmospheric Water Harvesting: A Multiscale Modeling Approach
Source: ACS Appl Mater Interfaces. 2026 Mar 4;18(10):15457–72. doi: 10.1021/acsami.5c25373 (PMC13006960; doi:10.1021/acsami.5c25373)
Supplement: Supplementary file 1 [file am5c25373_si_001.pdf]

Supporting Information

Trade-Off between Adsorption and Regeneration in Functional Metal-Organic Frameworks for Atmospheric Water Harvesting: A Multiscale Modeling Approach

Mehrzad Arjmandi[a], *Mohamed Khayet*[a,b*], *Takeshi Matsuura*[c]

[a]Department of Structure of Matter, Thermal Physics and Electronics, Faculty of Physics, University Complutense of Madrid, Avda. Complutense s/n, 28040 Madrid, Spain

[b]Madrid Institute for Advanced Studies of Water (IMDEA Water Institute), Avda. Punto Com N° 2, 28805 Alcalá de Henares, Madrid, Spain

[c]Department of Chemical and Biological Engineering, University of Ottawa, 161 Louis Pasteur Private, Ottawa, Ontario K1N 6N5, Canada

[*]Corresponding authors: khayetm@fis.ucm.es

Supplementary Figure S1

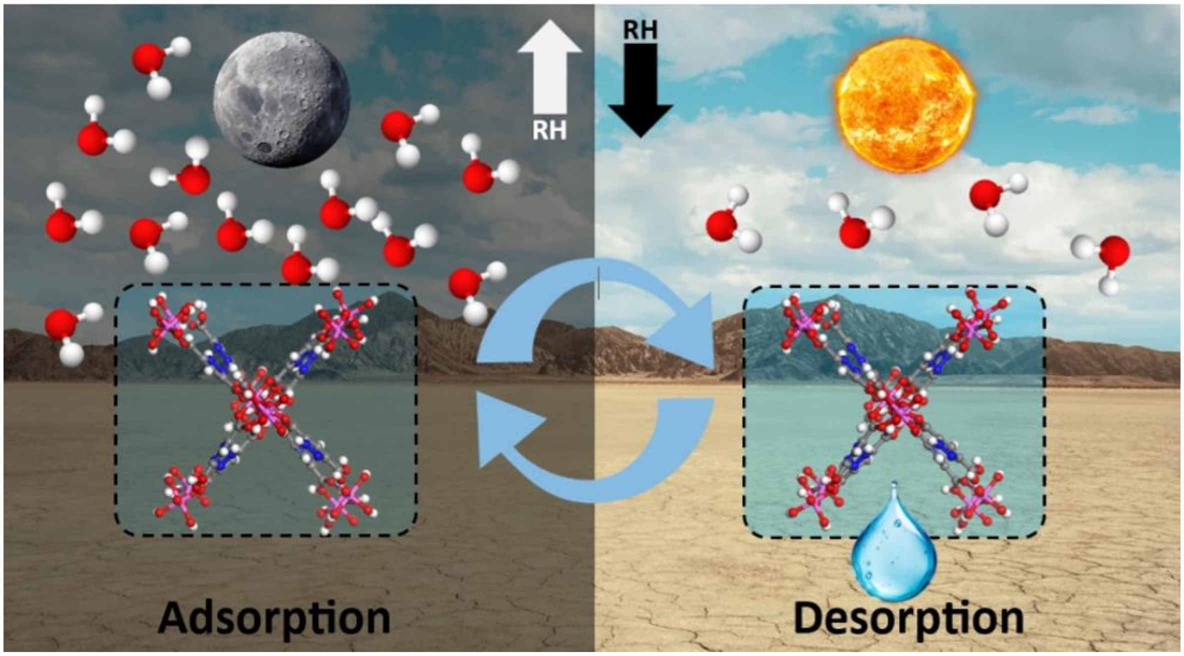

Figure S1. Schematic of the SAWH process: nighttime adsorption and daytime desorption of water vapor.

Supplementary Figure S2

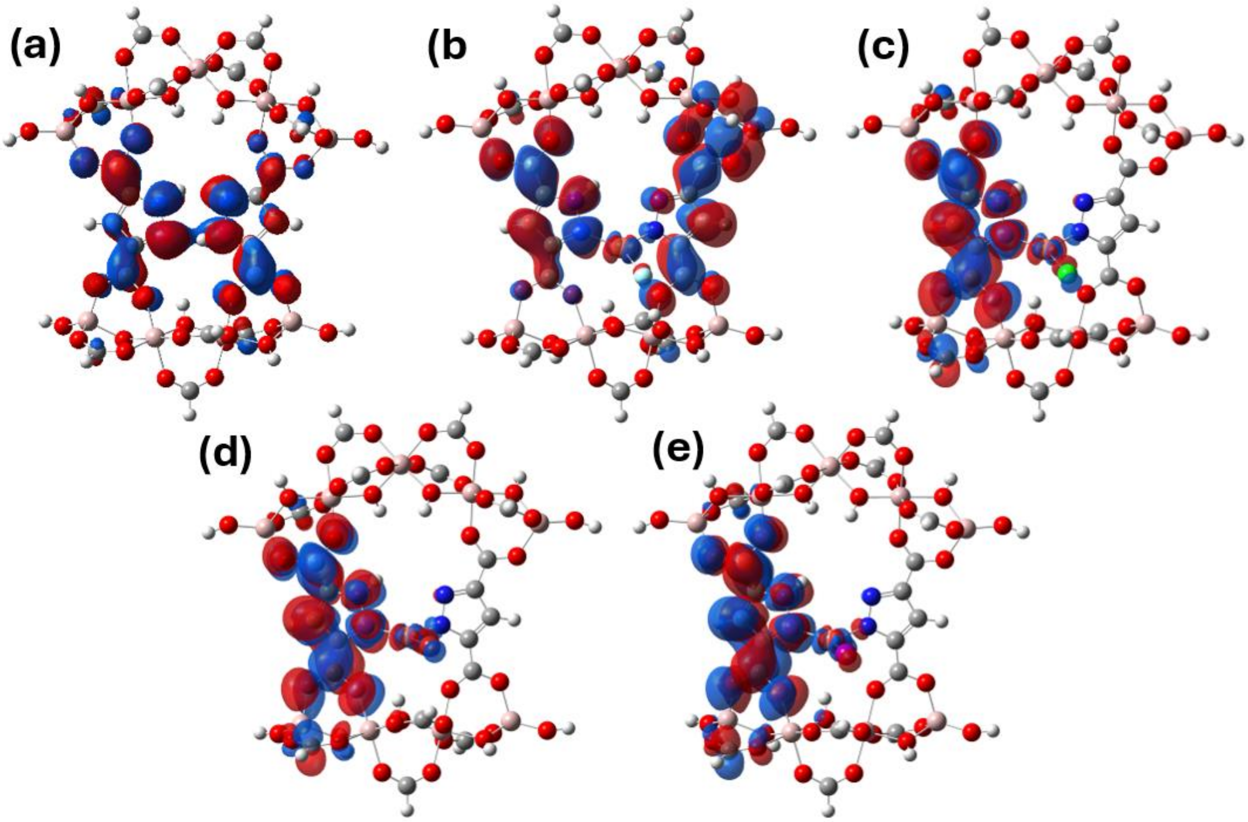

Figure S2. Spatial distribution of the HOMO orbitals for (a) pristine MOF-303, (b) Cu-F@MOF-303, (c) Cu-Cl@MOF-303, (d) Cu-Br@MOF-303, (e) Cu-I@MOF-303 systems before water adsorption.

Supplementary Figure S3

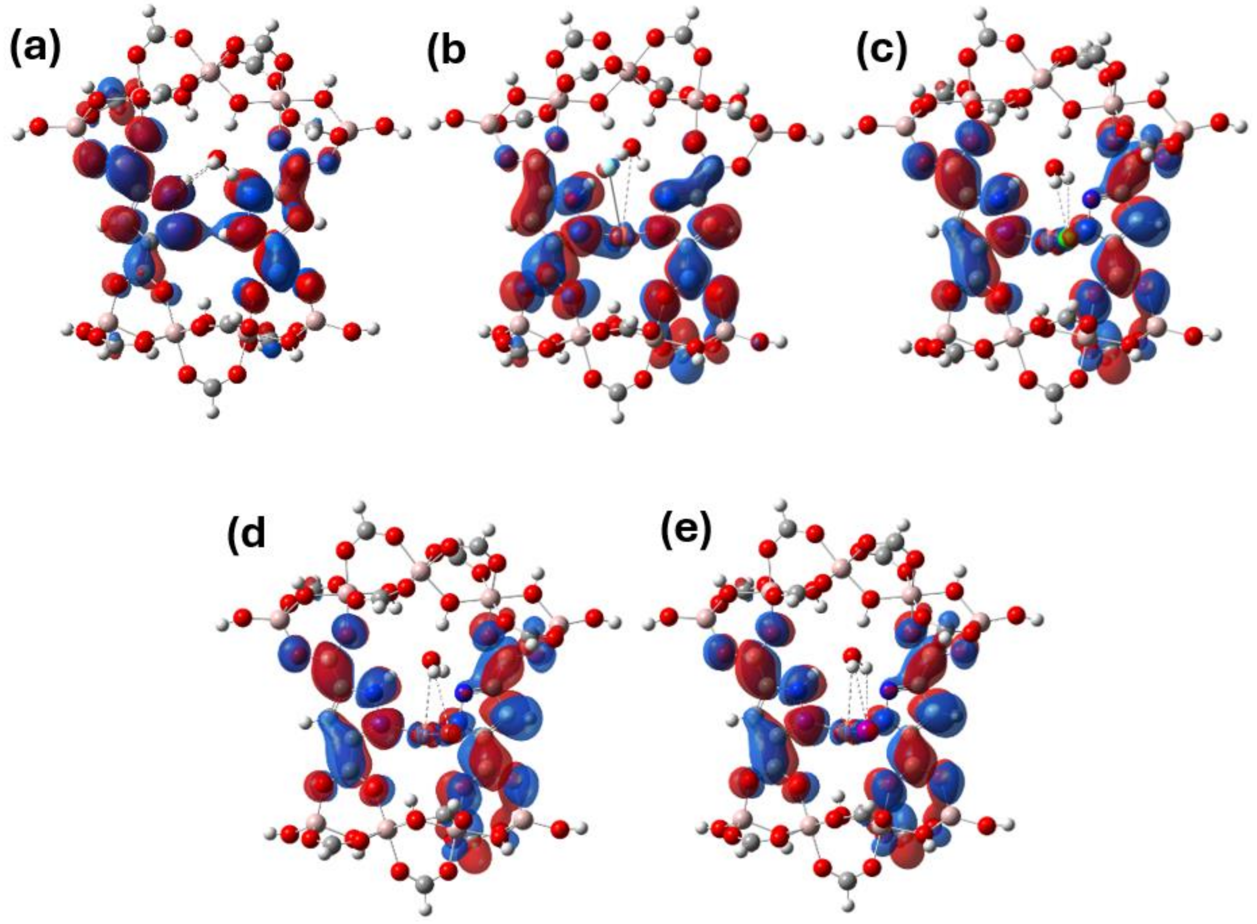

Figure S3. Spatial distribution of the HOMO orbitals for (a) pristine MOF-303, (b) Cu-F@MOF-303, (c) Cu-Cl@MOF-303, (d) Cu-Br@MOF-303, (e) Cu-I@MOF-303 systems after water adsorption.

Supplementary Figure S4

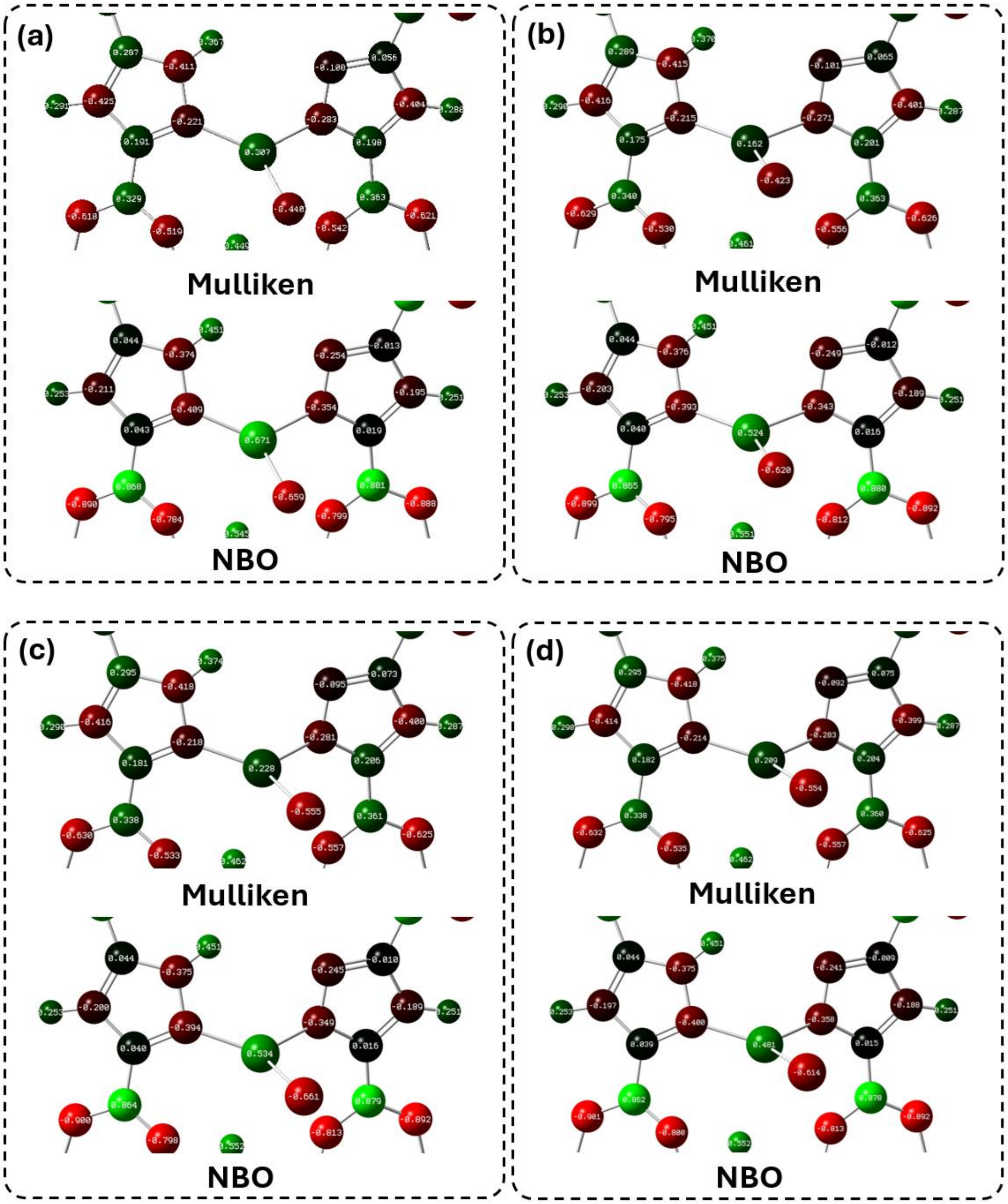

Figure S4. NBO and Mulliken charge distributions of atoms in (a) Cu-F@MOF-303, (b) Cu-Cl@MOF-303, (c) Cu-Br@MOF-303, and (d) Cu-I@MOF-303 systems.
